# Hydrothermally Synthesized Rare-Earth Oxides as Positive Electrodes for PVA/KOH Gel-Electrolyte Hybrid Supercapacitors

**DOI:** 10.3390/ma19194214

**Published:** 2026-10-03

**Authors:** Prasad E. Lokhande

**Affiliations:** Institute of Fundamental Technological Research, Polish Academy of Sciences, 02-106 Warsaw, Poland; plokh@ippt.pan.pl

**Keywords:** rare-earth oxides, CeO_2_, gel electrolyte, hybrid supercapacitor, pseudocapacitance, hydrothermal method

## Abstract

**Highlights:**

- Rare-earth oxides were hydrothermally synthesized and annealed.
- CeO_2_ delivered the highest specific capacitance among all investigated oxides.
- Y_2_O_3_ exhibited exceptional cycling stability during long-term electrochemical testing.
- A CeO_2_-based solid-state hybrid supercapacitor achieved 20 Wh kg^−1^ at 1000 W kg^−1^.

**Abstract:**

Rare-earth metal oxides are an exciting group of pseudocapacitive materials, but there are limited comparative studies on their electrochemical performance and the further fabrication of gel-electrolyte-based energy-storage devices. In the present study, CeO_2_, La_2_O_3_, Y_2_O_3_, Sm_2_O_3_, and Pr_2_O_3_ were prepared via the hydrothermal method and then subsequently annealed to study their supercapacitor applications. A crystallinity study carried out using XRD confirmed the formation of crystalline oxide phases, while FESEM and N_2_ adsorption–desorption analyses revealed oxide-dependent structural, surface, and porosity characteristics. Maximum specific capacitance was exhibited for CeO_2_, while Y_2_O_3_ exhibited exceptional cycling stability. The optimal values obtained from the CeO_2_ electrode were used to assemble a hybrid supercapacitor device with a PVA/KOH gel electrolyte to evaluate its practical applicability. The device achieved an energy density of 20 Wh kg^−1^ at a power density of 1000 W kg^−1^ and maintained 100% capacitance retention after 2000 cycles. In summary, each oxide had distinct advantages; CeO_2_ showed the best overall electrochemical response, signifying its potential in solid-state hybrid supercapacitors.

## 1. Introduction

Growing environmental degradation and rising global temperatures have intensified the shift away from fossil-based energy systems toward clean and renewable alternatives [1,2,3]. However, these intermittent renewable sources, particularly wind and solar, require energy-storage solutions to ensure reliable and constant power supply [4,5,6]. Among the various electrochemical storage systems under investigation, batteries, fuel cell, and supercapacitor technologies remain the most prominent [7,8,9]. However, the major attention is envisioned in the case of supercapacitors, due to their ability for high power output, rapid charges and discharges, long lifespans, and environmentally friendly nature [9,10,11]. The electrochemical performance of supercapacitors greatly depends on the physicochemical properties of both electrode and electrolyte materials [12,13,14,15]. Numerous electrode materials have been extensively investigated, including carbon-based materials, transition metal oxides, and conductive polymers [16,17,18]. Among these, transition metal oxides such as NiO, MnO_2_, Co_3_O_4_, Fe_2_O_3_, and CuO exhibit superior electrochemical performance due to their strong pseudocapacitive behavior, which arises from multiple oxidation states and highly reversible faradaic reactions [19,20,21]. With these results in mind, rare-earth metal oxides have recently become attractive materials for use as supercapacitor electrodes. Such oxides including CeO_2_, La_2_O_3_, Sm_2_O_3_, Pr_2_O_3_, Gd_2_O_3_, and Y_2_O_3_ have demonstrated noteworthy electrochemical activity in recent reports [22,23]. The charge-storage properties of rare-earth oxides are intimately related to their variable valence state properties, defect rich structures and oxygen vacancy mediated redox processes [24]. For instance, CeO_2_ exhibits a reversible Ce^3+^/Ce^4+^ transition that provides additional faradaic sites for charge storage [8,24,25]. Oxygen vacancies can further enhance ion transport and charge-transfer kinetics by facilitating surface and subsurface redox reactions. Nevertheless, the electrochemical response of each rare-earth oxide differs considerably depending on its electronic configuration, crystallinity, morphology, surface area, and defect concentration [22,23]. Joseph et al. [26] synthesized two-dimensional CeO_2_ using the solvothermal method and found it to be suitable as a supercapacitor electrode with a specific capacitance of 1801 F g^−1^ at a current density of 1 A g^−1^. Likewise, Yadav et al. [27] investigated La_2_O_3_ thin films using the chemical bath deposition technique and achieved a value of specific capacitance of 147 F g^−1^ with a scan rate of 5 mV s^−1^. Despite previous studies of individual rare-earth metal oxide, comparative studies would help to gain deeper insights into the fundamental properties of their electrodes and could be useful in rationally designing future hybrid or composite electrodes.

Recently, gel electrolytes have gained increased attention interest for electrochemical energy storage because they associate the ionic conductivity of liquid electrolytes with the improved safety, non-leakage characteristics, and mechanical integrity of solid-state systems [28]. In supercapacitors, gel electrolytes can enhance device stability, simplify packaging, and enable solid-state or quasi-solid-state operation, which is particularly important for compact and flexible energy-storage configurations. Among the available systems, PVA/KOH gel electrolytes are widely employed in alkaline supercapacitors because of their facile preparation, good ionic transport properties, and compatibility with pseudocapacitive electrode materials. These advantages make gel-electrolyte-based hybrid supercapacitors an important research direction for next-generation energy-storage devices [29,30].

In the present study, CeO_2_, La_2_O_3_, Y_2_O_3_, Sm_2_O_3_, and Pr_2_O_3_ were synthesized using a hydrothermal route followed by annealing. These oxides were systematically examined to assess their structural features, morphology, surface area, and porosity. Their electrochemical behavior was further evaluated through cyclic voltammetry, galvanostatic charge–discharge, and electrochemical impedance spectroscopy. Based on the comparative analysis, the most efficient material was selected for the fabrication of a hybrid gel-based supercapacitor device employing a PVA/KOH gel electrolyte. The device performance was assessed in terms of energy density and power density. The novelty of this work lies in synthesizing a series of rare-earth metal oxides under identical conditions, enabling a fair electrochemical comparison, and subsequently utilizing the best-performing oxide for practical device fabrication to demonstrate real-world applicability.

## 2. Materials and Methods

### 2.1. Materials

Cerium nitrate hexahydrate (Ce(NO_3_)_3_·6H_2_O), lanthanum nitrate hexahydrate (La(NO_3_)_3_·6H_2_O), praseodymium nitrate hexahydrate (Pr(NO_3_)_3_·6H_2_O), samarium nitrate hexahydrate (Sm(NO_3_)_3_·6H_2_O), and yttrium nitrate hexahydrate (Y(NO_3_)_3_·6H_2_O) served as the starting rare-earth salts. Hexamethylenetetramine (HMT) was employed to facilitate homogeneous precipitation during synthesis. All chemicals were procured from Sigma-Aldrich (Santiago, Chile) and used as received without further purification.

### 2.2. Rare-Earth Metal Oxide Preparation

**Figure 1:**
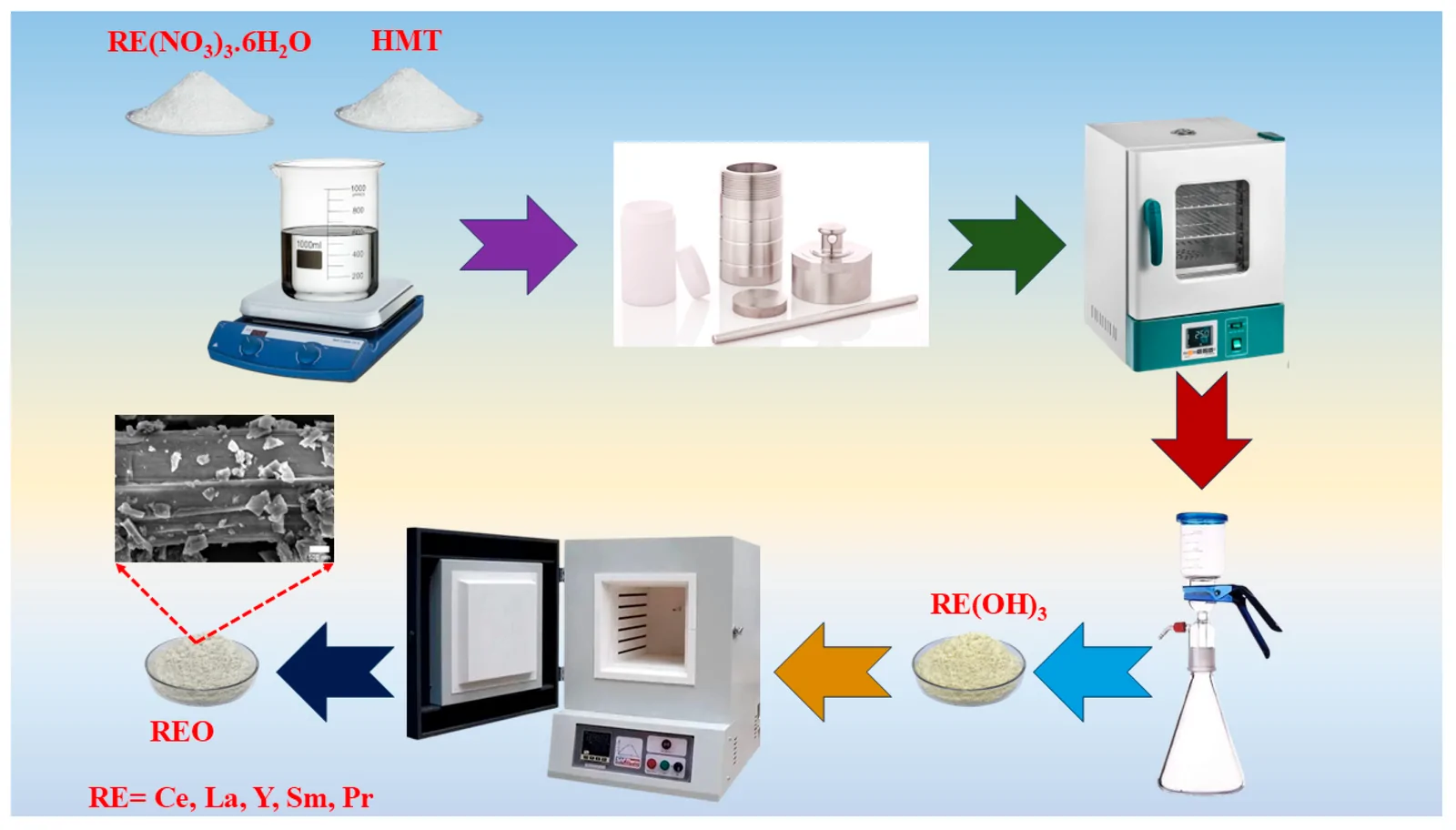
Schematic representation of hydrothermally synthesized rare-earth metal oxides.

All chemicals and reagents used in this study were employed as received without any additional purification. The nickel foam—used as both the substrate and current collector—was obtained from Xiamen Tob New Energy Technology Co., LTD., Xiamen, China. The rare-earth metal oxides were prepared via a hydrothermal process followed by thermal annealing. The precursor solution was prepared by dissolving 0.1 M Ce(NO_3_)_3_·6H_2_O in 50 mL of deionized water while stirring at room temperature, and 2 M hexamethylenetetramine (HMT) was also added to the precursor solution at room temperature. The mixed solution was then transferred to Teflon lined stainless-steel autoclave and heated at 120 °C for 4 h. Upon cooling to ambient temperature, the resulting precipitate was repeatedly washed with deionized water and ethanol and then dried for 12 h at 70 °C. The dried powder was subsequently calcined at 500 °C for 4 h to obtain crystalline CeO_2_. Figure 1 presents the step-by-step oxide synthesis route. The same synthesis procedure was applied to prepare La_2_O_3_, Y_2_O_3_, Sm_2_O_3_, and Pr_2_O_3_, with the cerium nitrate precursor replaced by the corresponding rare-earth nitrate salt.

### 2.3. Characterization

Various characterization techniques were employed to investigate the material properties of the synthesized samples. X-ray powder diffraction (XRD) with a Bruker D8 Advance equipped (Bruker AXS GmbH, Karlsruhe, Germany) with Cu Kα radiation (λ = 1.5406 Å) was used for structural analysis. The field emission scanning electron microscopy (FE-SEM, ZEISS, Carl Zeiss Microscopy GmbH, Jena, Germany) was used to study the surface morphology of the samples and to see details on their micro- and nanostructures. Fourier transform infrared spectroscopy (FTIR, PerkinElmer, PerkinElmer, Inc., Shelton, CT, USA) was used to identify the functional groups present in the materials. Surface area and porosity characteristics were evaluated via Brunauer–Emmett–Teller (BET) analysis, using a Nova 600 system from Anton Paar (Boynton Beach, FL, USA).

### 2.4. Electrode Fabrication

A three-electrode configuration was employed to evaluate the electrochemical performance of the individual electrodes, and the working electrodes were prepared using a slurry-casting approach. The slurry was formulated by mixing the synthesized oxide (active material), carbon black (conductive additive), and PVDF binder in an 85:10:5 weight ratio, dispersed in N-methyl-2-pyrrolidone (NMP). The mixture was stirred for 2 h to achieve uniform dispersion. This slurry was then applied onto pre-cut nickel foam pieces (1 × 2 cm^2^) to obtain a uniform coating, followed by drying at 70 °C for 4 h. Prior to deposition, the nickel foam was treated with dilute HCl to remove surface oxides and contaminants. The active mass loading, determined from the weight difference before and after coating.

The electrochemical performance of the prepared electrode was evaluated using a three-electrode configuration on a Gamry Interface 1010E system (Gamry Instruments, Warminster, PA, USA). A platinum wire and an Ag/AgCl electrode served as the counter and reference electrodes, respectively, while 3 M KOH was used as the electrolyte. Electrochemical impedance spectroscopy was performed over a frequency range of 100 kHz to 0.01 Hz. Cycling stability measurements were carried out at a current density of 10 A g^−1^. The specific capacitance was measured from the results obtained from cyclic voltammetry (CV) and galvanostatic charge–discharge (GCD) using the following equations:

(1) $$C_{s}=\frac{\int_{V1}^{V2}I\left(V\right)dV}{mv(V_{2}-V_{1})}$$

(2) $$C_{s}=\frac{I\Delta t}{m\Delta V}$$

where *I* represent the instantaneous current (A), *m* is the mass of the active material (g), *v* denotes the scan rate (mV·s^−1^), Δ*t* is the discharge time (s), and (Δ*V* = *V*_2_ − *V*_1_) corresponds to the applied potential window (*V*).

### 2.5. PVA-KOH Gel-Electrolyte Preparation

The solid-state PVA/KOH gel electrolyte was prepared using a solution-casting approach. First, 3 g of polyvinyl alcohol (PVA, Mw ≈ 89,000–98,000) was dissolved in 30 mL of deionized water. In a separate beaker, 3 g of potassium hydroxide (KOH) pellets were dissolved in deionized water, and this KOH solution was subsequently added to the PVA solution. The mixture was heated and stirred vigorously at 80 °C until a clear, homogeneous gel was obtained. The viscous gel was then cooled to room temperature and degassed under vacuum to remove trapped air bubbles before being poured onto a sterile Petri dish. Finally, the cast solution was left to solidify at room temperature for 24 h, forming a flexible, free-standing PVA–KOH gel-electrolyte membrane suitable for cell assembly.

### 2.6. Assembly of the CeO_2_//AC Hybrid Supercapacitor

A hybrid solid-state supercapacitor device was fabricated by using CeO_2_ as a positive electrode material, activated carbon as a negative electrode material, and PVA/KOH gel as an electrolyte. The charge-storage capacities of the positive and negative electrodes were optimized for the device through the mass balance. Then, the gel-electrolyte solution was added between the two electrodes, and the assembled device was allowed to stabilize prior to electrochemical measurements. The specific capacitance, energy density, and power density values of the hybrid device were obtained by using the following equations:

(3) $$C_{s}=\frac{I\Delta t}{m\Delta V}$$

(4) $$E=\frac{C_{s}V^{2}}{2\times 3.6}$$

(5) $$P=\frac{E\times 3600}{\Delta t}$$

where *C_s_* is the device-specific capacitance (Fg^−1^), *m* is the total mass of the electrode (g), *E* is the energy density (Wh kg^−1^), and *P* is the power density (W kg^−1^).

## 3. Results

**Figure 2:**
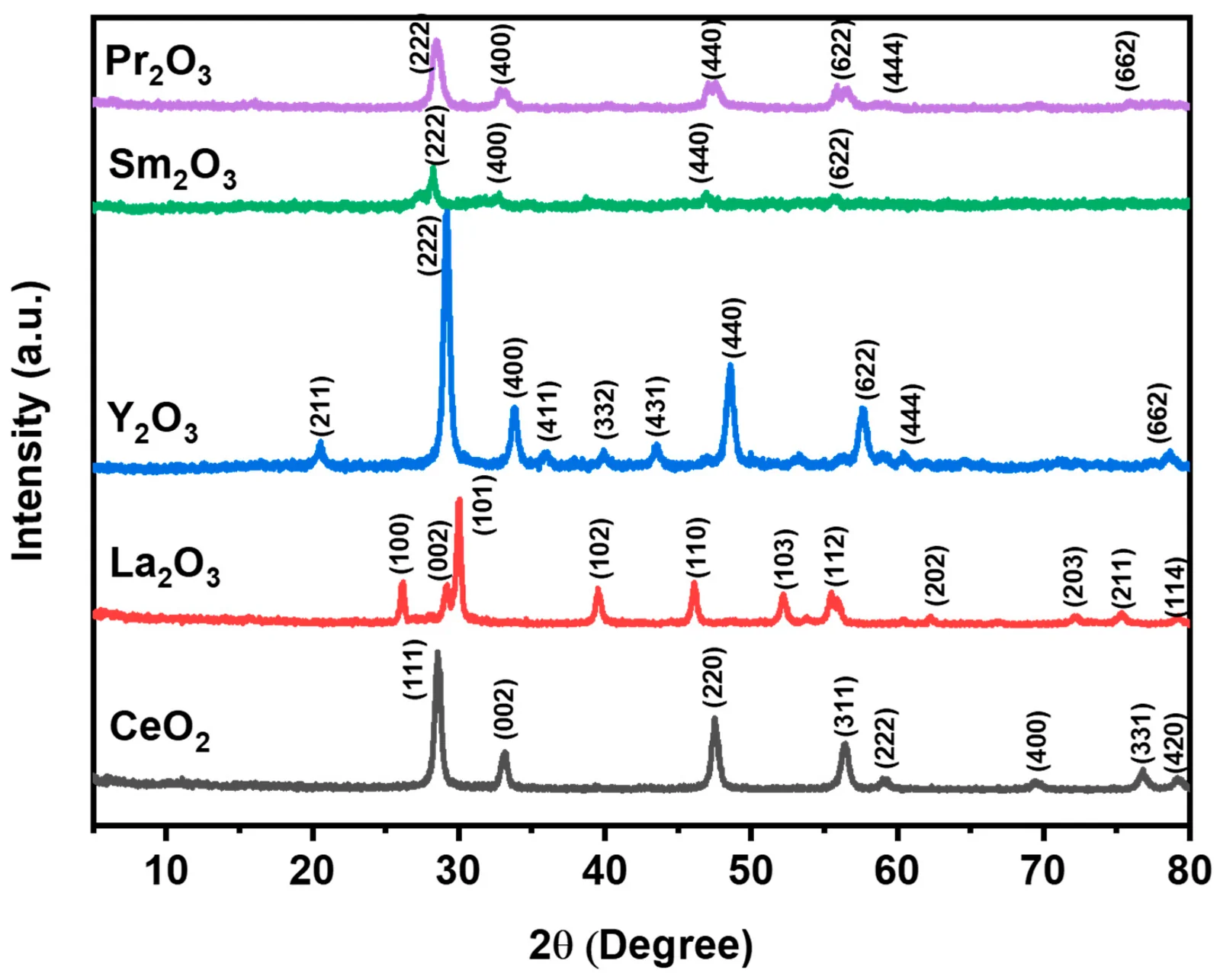
XRD peaks for the rare-earth metal oxides CeO_2_, La_2_O_3_, Y_2_O_3_, Sm_2_O_3_, and Pr_2_O_3_.

The crystal structures of the synthesized rare-earth oxides were characterized by X-ray diffraction, and the corresponding patterns are shown in Figure 2. CeO_2_ displayed well-defined reflections at 2θ values of 28.50°, 33.10°, 47.50°, 56.30°, 59.0°, 69.20°, 76.80°, and 78.90°, which correspond to the (111), (200), (220), (311), (222), (400), (331), and (420) planes, confirming the formation of cubic fluorite CeO_2_ in agreement with JCPDS 34 0394 [31]. La_2_O_3_ exhibited diffraction peaks at 26.1°, 29.1°, 30°, 39.5°, 46.1°, 52.2°, 55.5°, 62.2°, and 72.1°, corresponding to (100), (002), (101), (102), (110), (103), (112), (202), and (203) plane, consistent with the crystalline La_2_O_3_ phase reported in JCPDS 73 2141 [32]. The XRD pattern of Y_2_O_3_ showed characteristic peaks at 20.5°, 29.2°, 33.7°, 36.0°, 39.9°, 43.5°, 48.6°, 57.6°, and 60.3°, corresponding to the (211), (222), (400), (411), (332), (134), (440), (622), and (444) planes, matching JCPDS 88 1040 [33]. Sm_2_O_3_ presented prominent reflections at 18.7°, 28.2°, 32.7°, 46.9°, and 55.5°, indexed to the (211), (222), (400), (440), and (622) planes, confirming its crystalline nature in accordance with JCPDS 74 1807 [34]. Likewise, Pr_2_O_3_ exhibited distinct diffraction peaks characteristic of its crystalline phase, consistent with JCPDS 01 082 1401 [35].

The crystallite sizes of CeO_2_, La_2_O_3_, Pr_2_O_3_, Sm_2_O_3_, and Y_2_O_3_ were estimated from the XRD peak broadening using the Debye–Scherrer relation. Based on the measured peak widths, the apparent crystallite sizes were approximately 15 nm for CeO_2_, 21 nm for La_2_O_3_, 12 nm for Pr_2_O_3_, 20 nm for Sm_2_O_3_, and 15 nm for Y_2_O_3_. The noticeable broadening of the diffraction peaks supports the nanocrystalline nature of the synthesized oxides. These values represent coherent diffraction domain sizes rather than actual particle dimensions, and, since instrumental broadening was not separately corrected, they should be considered approximate Scherrer estimates.

**Figure 3:**
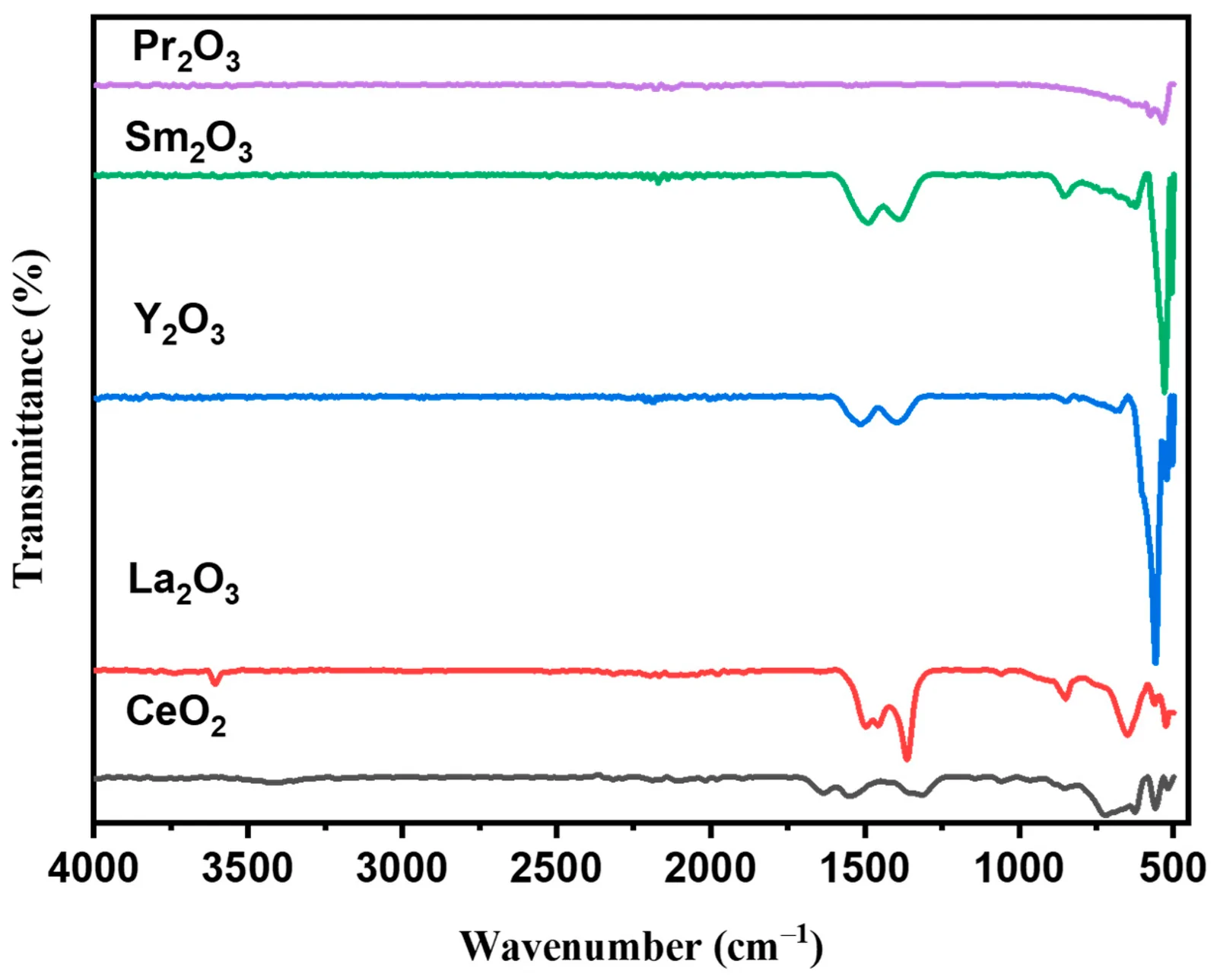
FTIR peaks for the rare-earth metal oxides CeO_2_, La_2_O_3_, Y_2_O_3_, Sm_2_O_3_, and Pr_2_O_3_.

The bonding features and surface functional groups of the synthesized rare-earth oxides were analyzed using FTIR spectroscopy. The FTIR spectra obtained from the prepared samples are presented in Figure 3. The high-intensity absorption band around 500–600 cm^−1^ was observed in all the samples, indicating the presence of metal–oxygen (M–O) stretching vibrations. A secondary band that appeared around 842–849 cm^−1^ in all materials except Pr_2_O_3_ may be associated with M–O–M bridging vibrations or residual carbonate related modes. The peaks at 1370 and 1516 cm^−1^ are attributed to surface adsorbed carbonate species, which may be from the adsorption of CO_2_ from the atmosphere or from the remnants of the decomposition of the precursor. In addition, CeO_2_ showed a wide absorption feature around 3400 cm^−1^, which could be related to O–H stretching vibrations, suggesting the presence of adsorbed moisture or surface hydroxyl groups unique to this sample.

**Figure 4:**
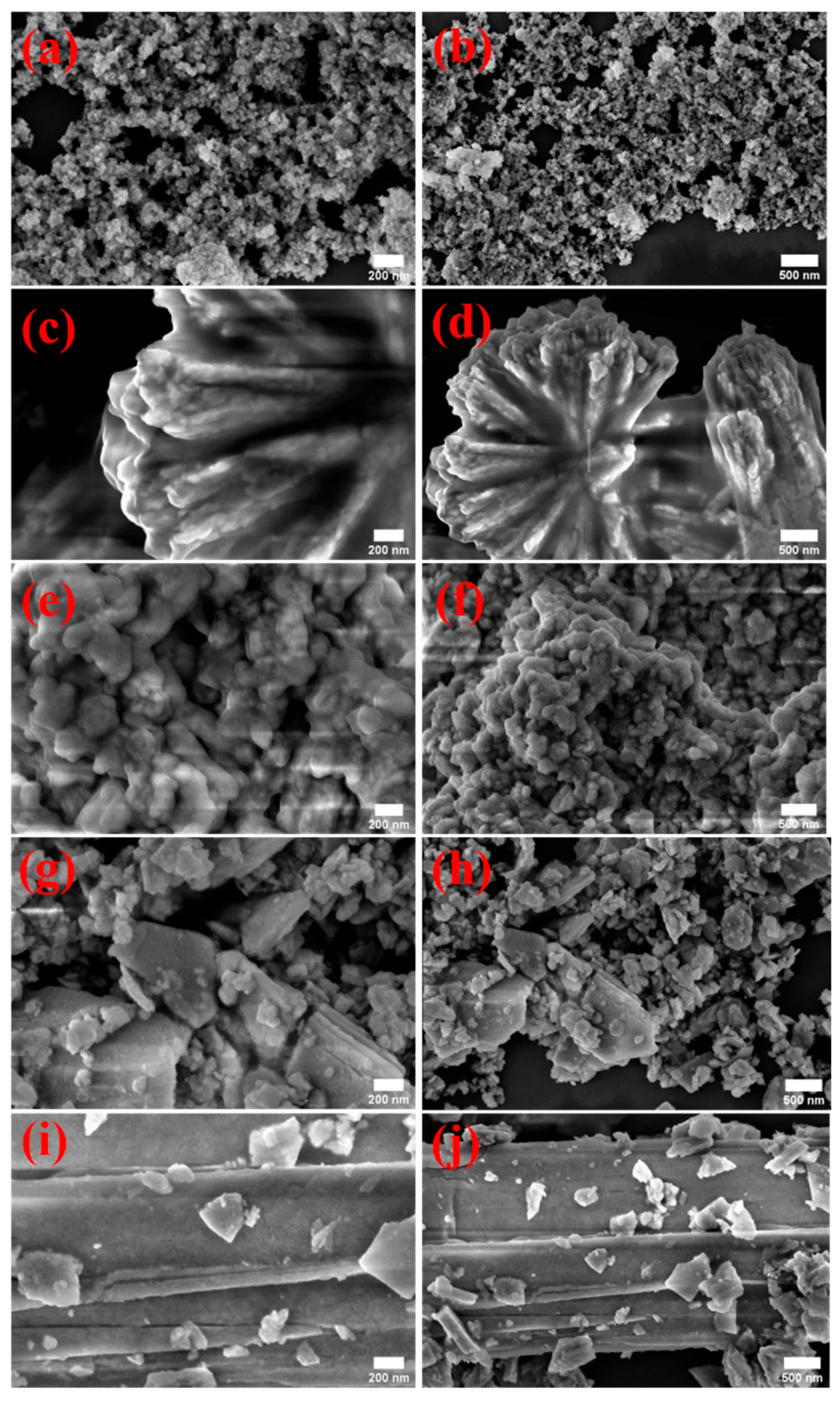
FESEM images for rare-earth metal oxides: (**a**,**b**) CeO_2_, (**c**,**d**) La_2_O_3_, (**e**,**f**) Y_2_O_3_, (**g**,**h**) Sm_2_O_3_, and (**i**,**j**) Pr_2_O_3_.

A morphological study of rare-earth oxides were carried out using FESEM, and the obtained morphologies are presented in Figure 4. CeO_2_ (Figure 4a,b) displays a nanosphere-type architecture, where interconnected spherical particles cluster together to form a porous network capable of offering numerous accessible active sites and facilitating electrolyte diffusion. La_2_O_3_ (Figure 4c,d) shows well-defined nanorod structures that assemble into flower-like arrangements. Y_2_O_3_ (Figure 4e,f) exhibits a distinct nanoplate morphology with an open surface layout that may enhance ion transport. Sm_2_O_3_ (Figure 4g,h) presents a layered arrangement comprising stacked nanoplates, potentially enabling efficient ion pathways. Pr_2_O_3_ (Figure 4i,j) features a hierarchical platelet-based structure, where larger plates serve as a base for the attachment of smaller plate-like units. Overall, the observed morphological variations indicate that the hydrothermal synthesis route produced oxide-specific nanostructures, each of which may influence electrolyte penetration, active site exposure, ion diffusion, and, ultimately, the electrochemical behavior of the resulting electrodes.

The surface chemistry and oxidation states of the CeO_2_ sample were examined using XPS, as shown in Figure S1. Overall, the XPS analysis confirms that Ce^4+^ is the predominant oxidation state in the sample, as reflected by the characteristic six-component Ce^4+^ multiplet and the intense u‴ peak near 916.4 eV. The weaker Ce^3+^ contributions point to limited surface reduction, resulting in a mixed-valence Ce^3+^/Ce^4+^ environment [14,36,37]. Together with the surface-related O 1s features, the results suggest the presence of a moderately defect-rich CeO_2−x_ surface and highlight the redox-active nature of the material.

**Figure 5:**
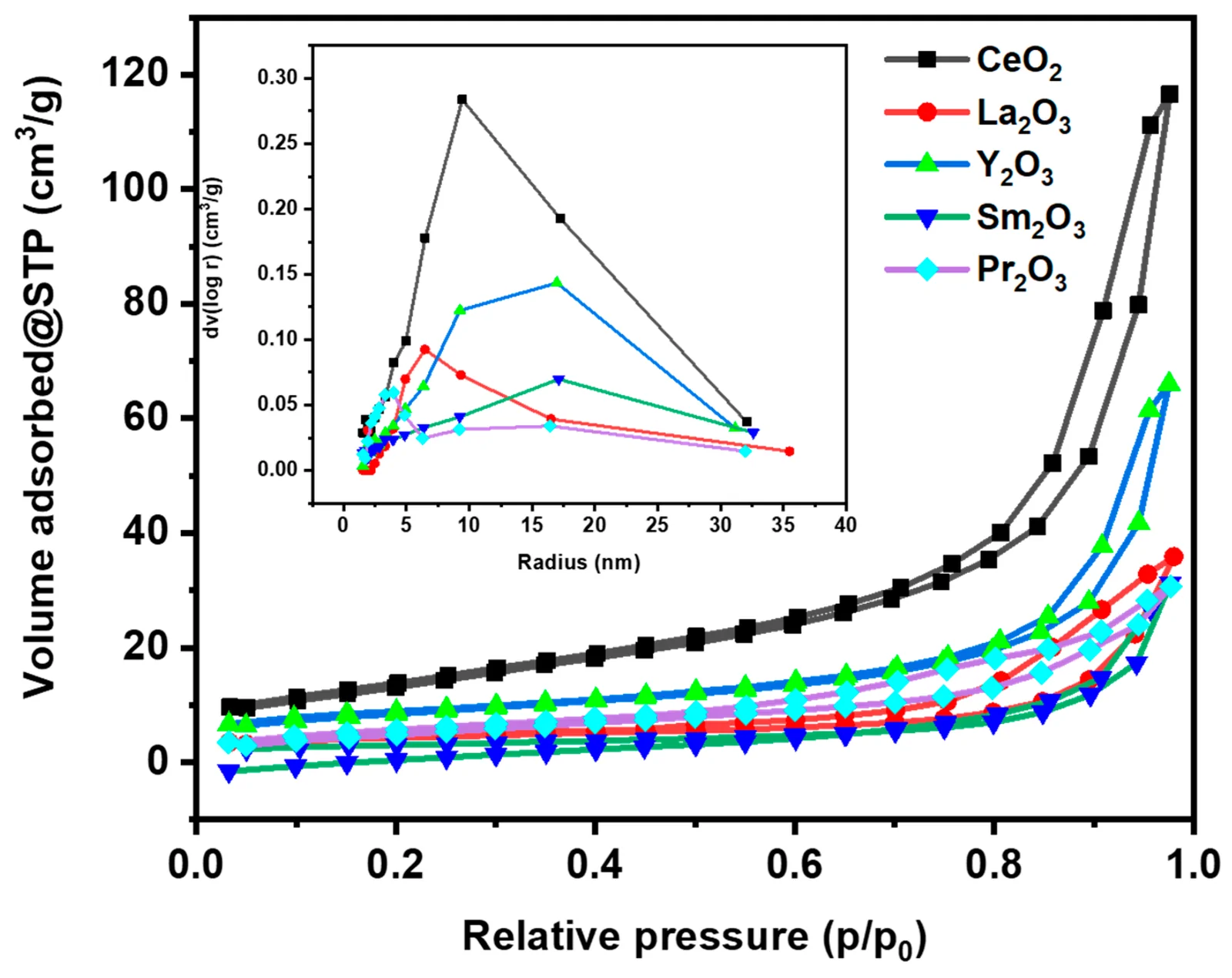
N_2_ isotherms for rare-earth metal oxides CeO_2_, La_2_O_3_, Y_2_O_3_, Sm_2_O_3_, and Pr_2_O_3_ (inside: pore-size distribution of CeO_2_, La_2_O_3_, Y_2_O_3_, Sm_2_O_3_, and Pr_2_O_3_).

The electrochemical behavior of the electrode materials is significantly influenced by their surface and porosity properties. To evaluate these properties, the nitrogen adsorption–desorption isotherms were recorded, as shown in Figure 5, and the textural parameters were extracted using BET and BJH analyses. Among the synthesized oxides, CeO_2_ exhibited the largest specific surface area (46.9 m^2^ g^−1^), trailed by Y_2_O_3_ (22.5 m^2^ g^−1^), Pr_2_O_3_ (18.6 m^2^ g^−1^), La_2_O_3_ (15.8 m^2^ g^−1^), and Sm_2_O_3_ (13.6 m^2^ g^−1^). This trend aligns with the FESEM observations, where CeO_2_ showed an interconnected nanosphere-type morphology that naturally provides greater surface exposure. The structural characteristics are beneficial for electrochemical applications since they increase the number of active sites and promote the infiltration of the electrolyte and ion transport. BJH pore-size analysis displayed average pore radii of 9.4 nm for CeO_2_, while La_2_O_3_, Y_2_O_3_, Sm_2_O_3_, and Pr_2_O_3_ exhibited values of 1.9, 9.2, 1.7, and 3.3 nm, respectively. These results indicate that most of the synthesized oxides possess mesoporous characteristics, which are favorable for efficient ion movement during charge–discharge processes. The combination of high surface area and mesoporosity in CeO_2_ is expected to significantly enhance its capacitive behavior.

**Figure 6:**
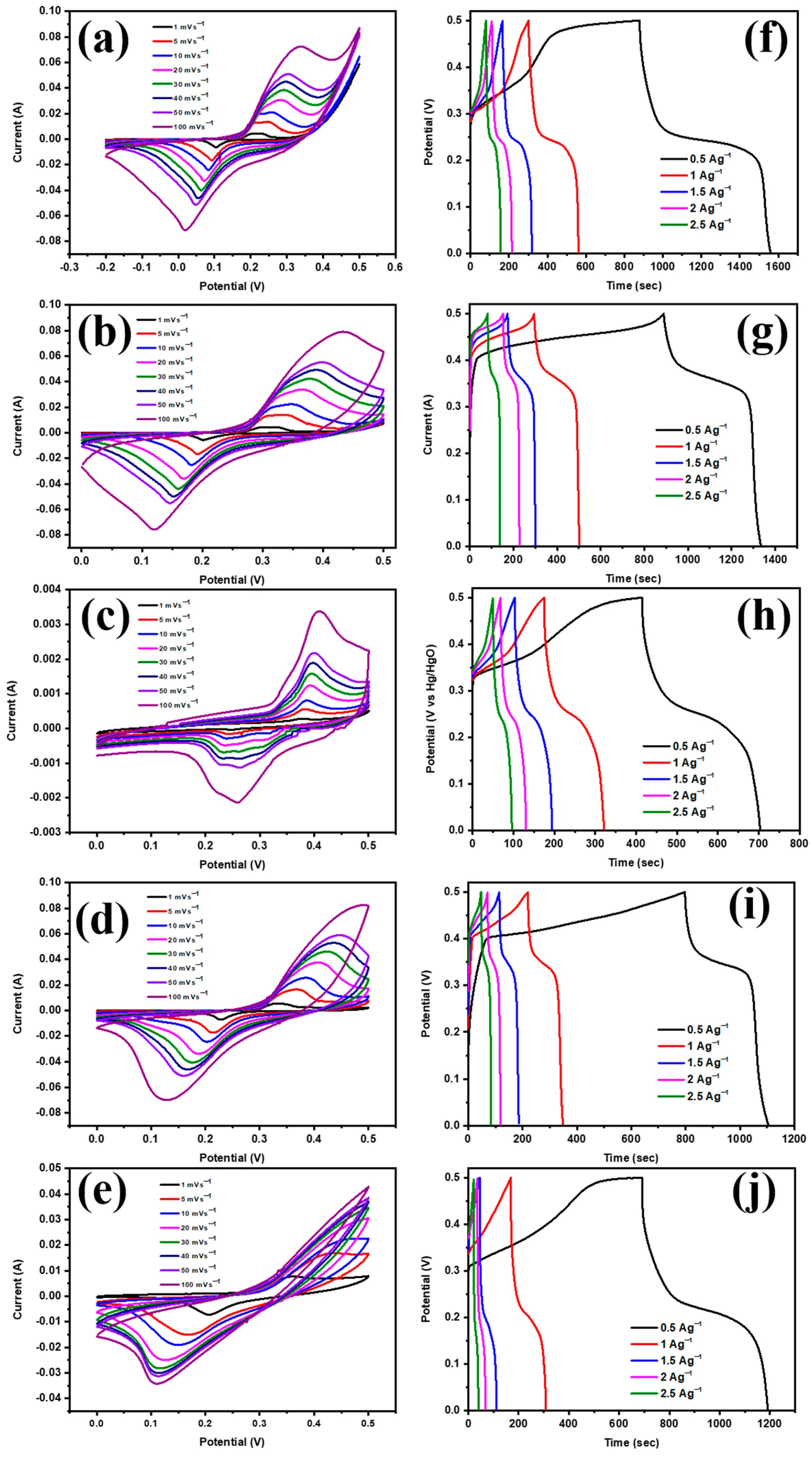
(**a**–**e**) CV curve at scan rate of 1, 5, 10, 20, 30, 40, 50 and 100 mVs^−1^ (**f**–**j**) GCD profile at current densities of 0.5, 1, 1.5, 2, 2.5 Ag^−1^ for CeO_2,_ La_2_O_3_, Y_2_O_3_, Sm_2_O_3_, and Pr_2_O_3_.

The electrochemical activity of the prepared rare-earth oxides were assessed under a conventional three electrode configuration in 3 M KOH. The active material deposited on the pre-treated nickel foam was used as the working electrode, and a platinum wire as the counter electrode. CV curves of all the samples were recorded under a scan rate from 1 to 100 mV s^−1^ in the voltage range of 0.0 to 0.5 V as shown in Figure 6a–e. Different redox signatures were found for each of the oxides, confirming redox behavior controlled by the reversible faradaic reactions. By increasing the scan rate, the anodic peaks moved to more positive potentials, while the cathodic peaks moved towards more negative potentials due to polarization effects and diffusion limited ion transport at higher sweep speeds. Among the investigated electrodes, CeO_2_ delivered the highest specific capacitance of 535 F g^−1^ at 1 mV s^−1^, followed by Pr_2_O_3_ (476 F g^−1^), Sm_2_O_3_ (393 F g^−1^), La_2_O_3_ (277 F g^−1^), and Y_2_O_3_ (239 F g^−1^). The superior performance of CeO_2_ is attributed to its comparatively larger surface area, porous nanosphere morphology, abundant electroactive sites, oxygen-vacancy-rich structure, and reversible Ce^3+^/Ce^4+^ transitions, all of which promote efficient ion diffusion and charge-transfer kinetics also demonstrated in XPS results.

Additional insight into the charge-storage mechanism was obtained from the GCD profiles shown in Figure 6f–j. All electrodes exhibited nonlinear charge–discharge curves with partial symmetry, a characteristic response of pseudocapacitive systems in which faradaic reactions dominate the energy-storage process. Consistent with the CV findings, CeO_2_ delivered the highest specific capacitance, reaching 684 F g^−1^ at 0.5 A g^−1^, followed by Pr_2_O_3_ (501 F g^−1^), Sm_2_O_3_ (445 F g^−1^), La_2_O_3_ (307 F g^−1^), and Y_2_O_3_ (288 F g^−1^). The remarkable performance of CeO_2_ arises from its interconnected porous nanosphere network, which offers a large accessible surface and numerous electroactive sites. This structural arrangement facilitates rapid electrolyte infiltration and short ion diffusion pathways, enabling efficient charge storage throughout the electrode. These combined structural and redox advantages account for the significantly enhanced capacitance and superior energy-storage capability of CeO_2_ relative to the other rare-earth oxides. Further kinetic partitioning of the charge storage, evaluated using the Dunn model, is presented in Figure S2, highlighting the distribution of capacitive and diffusion-controlled contributions.

**Figure 7:**
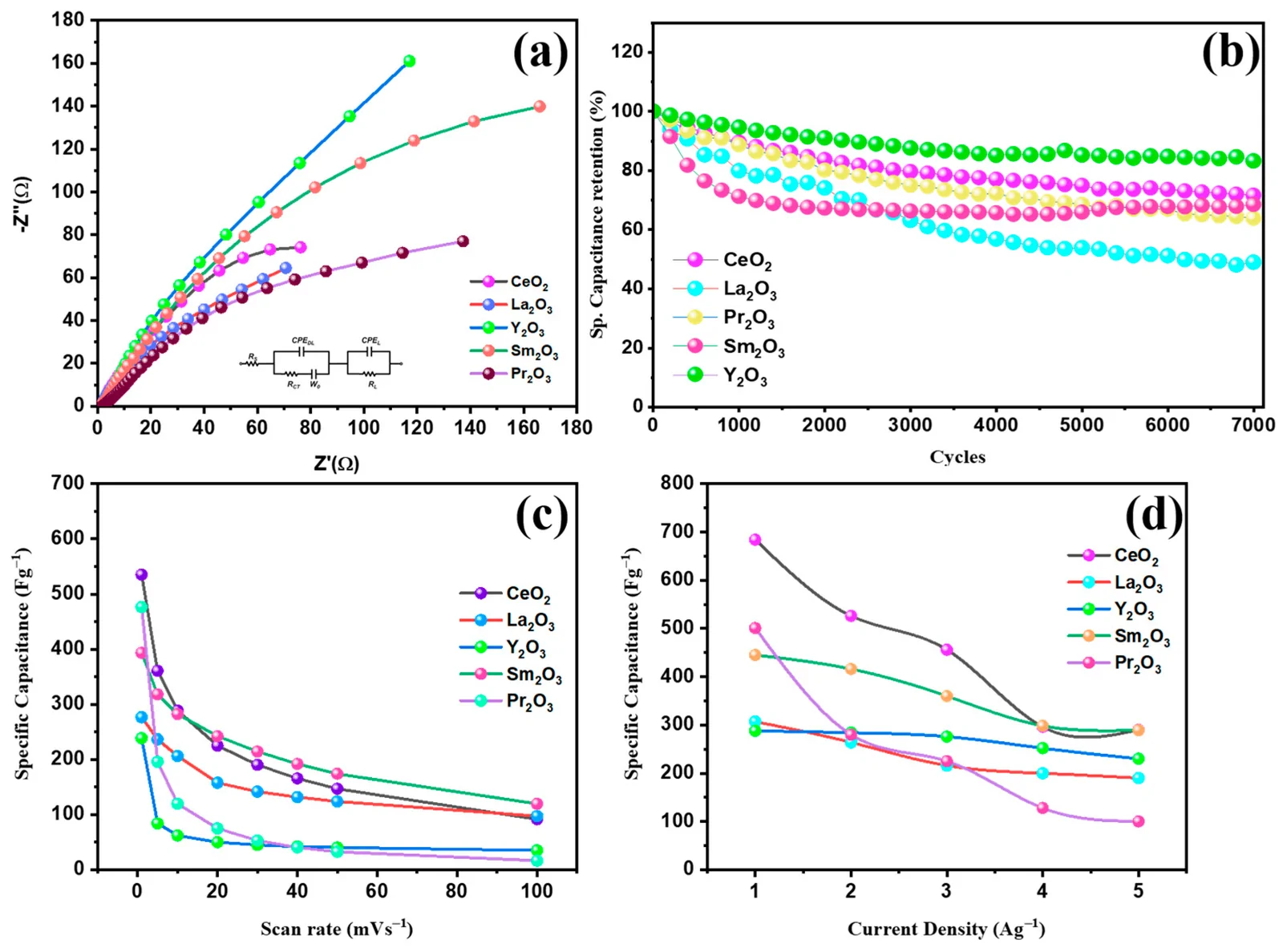
(**a**) Nyquist plot (**b**) Cyclic life test at 7000 cycles (**c**) Rate dependent performance at multiple scan rates (**d**) Rate dependent performance from GCD results for CeO_2_, La_2_O_3_, Y_2_O_3_, Sm_2_O_3_ and Pr_2_O_3_.

The resistive and interfacial characteristics of the prepared samples have been analyzed using the electrochemical impedance spectroscopy technique and the respective Nyquist plots are shown in Figure 7a. The solution resistance (R_s_) deduced from the high frequency intercept of the real axis (Z′) reflects the combined contributions of the electrolyte, the contact between the current collector and the electrode. The charge-transfer resistance (R_ct_), estimated from the semicircle diameter in the high frequency region, indicates the kinetics of faradaic reactions occurring at the electrode–electrolyte interface. The smaller semicircle indicating a decrease in R_ct_ which could correspond to a faster interfacial charge transfer and effective utilization of the electroactive sites.

Among all materials, CeO_2_ exhibited the lowest R_s_ value of 0.8 Ω, slightly below those of La_2_O_3_ (0.83 Ω), Sm_2_O_3_ (1.0 Ω), Y_2_O_3_ (1.5 Ω), and Pr_2_O_3_ (1.7 Ω). The reduced R_s_ for CeO_2_ suggests minimal ohmic losses and strong electrical coupling between the active layer and the nickel foam substrate, consistent with its superior capacitive behavior observed in CV and GCD measurements. Its favorable impedance response can be attributed to the porous nanosphere morphology, larger accessible surface area, and higher density of electroactive sites, all of which facilitate electrolyte diffusion and charge transport. The long-term cycling behavior of the rare-earth oxide electrodes were evaluated through continuous charge–discharge testing over 7000 cycles, as shown in Figure 7b.

Among all materials, Y_2_O_3_ exhibited the highest capacitance retention of 82%, outperforming CeO_2_ (72%), Sm_2_O_3_ (68%), Pr_2_O_3_ (64%), and La_2_O_3_ (49%). The strong durability of Y_2_O_3_ indicates excellent structural integrity and resistance to degradation during repeated electrochemical operation. In contrast, the comparatively low retention of La_2_O_3_ suggests a greater susceptibility to structural deterioration, diminished electrical pathways, or loss of active sites during prolonged cycling. The intermediate retention values observed for CeO_2_, Sm_2_O_3_, and Pr_2_O_3_ reflect partial preservation of their electrochemical activity, although some decline in performance occurs with extended use. The variations in cycling stability among the rare-earth oxide electrodes can be attributed to differences in crystallinity, structural resilience, ion transport pathways, and the mechanical robustness of the electrode–electrolyte interface. These findings highlight that material specific characteristics—particularly phase stability and resistance to structural deterioration under repeated cycling—are decisive factors governing long-term electrochemical performance. Among the investigated oxides, Y_2_O_3_ exhibited the most stable behavior, maintaining 82% of its initial capacitance after 7000 cycles.

The rate dependent performance of the rare-earth oxide electrodes was investigated by CV and GCD analysis which can be seen in Figure 7c,d. Rate capability reflects the extent to which an electrode can retain its capacitance under progressively higher scan rates or current densities, a critical parameter for high power energy-storage systems. Among all materials, CeO_2_ exhibited the most robust rate performance, maintaining a larger proportion of its initial capacitance under rapid charging conditions. This behavior signifies that CeO_2_ enables fast ion transport and supports efficient charge-transfer kinetics. Its lower solution resistance, as revealed by EIS analysis, also helps minimize ohmic losses and suppress polarization during high-rate operation. In contrast, the other oxides showed more pronounced capacitance decay at elevated rates, likely due to slower ion diffusion and restricted access to internal electroactive regions. Summarizing the results, it can be concluded that CeO_2_ possesses the most optimal high-rate response among the investigated materials, demonstrating its potential application for systems with rapid charge–discharge requirements.

**Figure 8:**
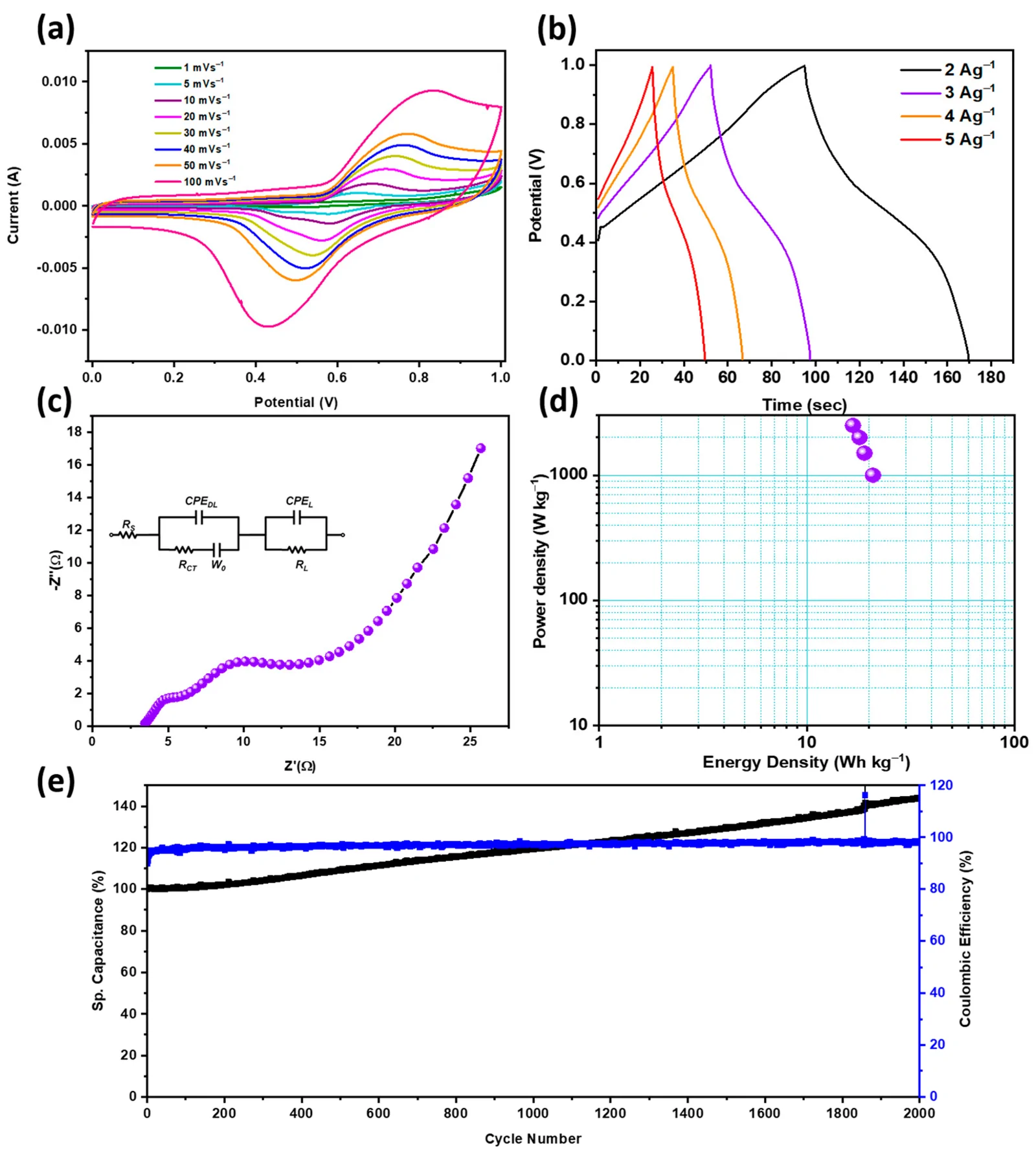
Electrochemical performance analysis of the CeO_2_//AC hybrid device: (**a**) CV curves at different scan rates; (**b**) GCD profiles at various current densities; (**c**) Nyquist plot obtained from EIS measurements; (**d**) Ragone plot derived from energy and power density values; and (**e**) cycling stability over 2000 charge–discharge cycles.

The practical applicability of the synthesized material was assessed by fabricating a solid-state hybrid supercapacitor, in which a PVA/KOH polymer gel acted as the electrolyte. The most efficient CeO_2_ was used as the positive electrode and the activated carbon as the negative electrode. The assembled device was evaluated in a two-electrode configuration using CV, GCD, and EIS techniques. The CV curve in Figure 8a exhibits a distinct hybrid charge-storage response within the operating potential window of 0.00–1.00 V. The obtained CV curve demonstrate the redox behavior dominance. GCD profiles recorded at various current densities, shown in Figure 8b, further validate the hybrid behavior, consistent with the CV observations. The obtained GCD profile demonstrates the nonlinear symmetric triangular profile which indicate the redo behavior aligned with CV result. The device achieved a maximum specific capacitance of 150 F g^−1^ at 1 A g^−1^, which leads to maximum energy density of 20 Wh kg^−1^ and maximum power density of 1000 W kg^−1^. Impedance analysis, presented in Figure 8c, reveals low solution resistance along with a moderately higher charge-transfer resistance, which is expected due to the gel-based electrolyte. The Ragone plot in Figure 8d, constructed from energy and power density values at different current loads, shows a maximum energy density of 20 Wh kg^−1^ at 1000 W kg^−1^, while the highest power density of 2500 W kg^−1^ was achieved at elevated current density. Cycling stability over 2000 charge–discharge cycles (Figure 8e) demonstrated nearly complete capacitance retention, confirming the mechanical and electrochemical robustness of the device. It is concluded that both the electrode-level and device-level results unequivocally indicate that CeO_2_ is a very promising material for advanced energy-storage devices.

## 4. Conclusions

A series of rare-earth metal oxides—CeO_2_, La_2_O_3_, Y_2_O_3_, Sm_2_O_3_, and Pr_2_O_3_—was effectively synthesized via a hydrothermal method followed by thermal annealing for applications as supercapacitor electrode materials. Structural analyses verified the development of the desired crystalline phases, while morphological studies revealed distinct micro and nanostructured features conducive to efficient charge storage. Comparative electrochemical evaluation showed that each oxide exhibited characteristic advantages determined by its intrinsic physicochemical properties, surface architecture, and charge-transfer behavior. Among the investigated materials, CeO_2_ delivered the maximum specific capacitance of 684 F g^−1^ at 0.5 A g^−1^, along with the lowest solution resistance and excellent rate capability. These superior attributes arise from its high surface area, porous nanosphere morphology, abundant electroactive sites, and reversible Ce^3+^/Ce^4+^ redox activity, all of which facilitate rapid transport of ion and efficient charge-transfer kinetics. In contrast, Y_2_O_3_ displayed the most stable long-term cycling behavior, retaining 82% of its initial capacitance after 7000 cycles, indicating strong structural durability under extended electrochemical operation. The solid-state hybrid supercapacitor with CeO_2_ delivered a significant energy density of 20 Wh kg^−1^, with a maximum power density of 1000 W kg^−1^ while maintaining nearly full capacitance retention after 2000 cycles, demonstrating excellent device level stability. Overall, the results highlight CeO_2_ as the most balanced and high performing electrode material among the studied oxides, while Y_2_O_3_ excels in long-term durability. The results highlighted the promise of rare-earth metal oxides to become a promising and multifunctional material for next-generation high-performance supercapacitor technologies.

## Data Availability

The original contributions presented in this study are included in the article and the supplementary material. Further inquiries can be directed to the corresponding author.

## Supplementary Materials

The following supporting information can be downloaded at: https://www.mdpi.com/article/10.3390/ma19194214/s1, Figure S1: XPS analysis of CeO_2_. (a) Core spectra of Ce 3d. (b) Core spectra of O 1s; Figure S2: Relative contributions of different energy storage mechanisms as a function of the scan rate; Table S1: Comparative summary of the electrochemical performance of synthesized materials with the reported literature.
