# Supplementary material for: Hydrothermally Synthesized Rare-Earth Oxides as Positive Electrodes for PVA/KOH Gel-Electrolyte Hybrid Supercapacitors
Source: Materials (Basel). 2026 Oct 3;19(19):4214. doi: 10.3390/ma19194214 (PMC13666230; doi:10.3390/ma19194214)
Supplement: Supplementary file 1 [file materials-19-04214-s001.zip › materials-4585179-supplementary.pdf]

Supporting Information for

Hydrothermally Synthesized Rare-Earth Oxides as Positive Electrodes for PVA/KOH Gel-Electrolyte Hybrid Supercapacitors

Prasad E. Lokhande

Institute of Fundamental Technological Research, Polish Academy of Sciences, 02-106 Warsaw, Poland; plokh@ippt.pan.pl

1. XPS study of CeO_2

The surface chemistry and oxidation states of the CeO_2 sample were examined using X-ray photoelectron spectroscopy (XPS). The high-resolution Ce 3d spectrum displays the typical multiplet pattern of cerium oxide, which arises from spin–orbit splitting and the different final-state configurations associated with Ce^{4+} and Ce^{3+}. After charge correction, the major Ce^{4+} contributions appear near 882.2, 888.7, 898.0, 900.6, 907.3, and 916.4 eV, corresponding to the well-known v, v″, v‴, u, u″, and u‴ features. The strong u‴ peak at around 916.4 eV is a characteristic signature of Ce^{4+} in CeO_2. Further to the dominant Ce^{4+} signals, additional intensity is observed in the regions typically associated with Ce^{3+}, particularly around 880–881, 885–886, 899, and 903–904 eV. These features correspond to the v^0, v′, u^0, and u′ states. The simultaneous presence of Ce^{3+} and Ce^{4+} indicates a partial reduction in Ce^{4+} near the surface, suggesting a defect-rich or slightly non-stoichiometric CeO_{2-x} structure.

The O 1s spectrum contains a main peak at approximately 529.0 eV, assigned to lattice O^{2-} in the Ce–O–Ce network. A broader component near 531.1 eV is attributed to oxygen species in modified surface environments, such as hydroxyl groups, carbonates, adsorbed oxygen, or oxygen associated with under-coordinated or defective sites. A higher-binding-energy feature around 535 eV likely arises from weakly adsorbed surface species or an energy-loss process rather than a distinct lattice oxygen state. It is important to note that the 531.1 eV component should not be directly interpreted as an "oxygen vacancy" signal, since a vacancy does not contain oxygen and therefore cannot produce an O 1s peak. Previous XPS studies on ceria have shown that hydroxyls, carbonates, and other surface oxygen species contribute significantly in this region, while the evolution of Ce^{3+} in the Ce 3d spectrum provides more reliable evidence of reduction. Thus, the combination of Ce^{3+} features in the Ce 3d spectrum and the higher-binding-energy O 1s component is more appropriately viewed as an indication of a chemically heterogeneous and partially reduced CeO_2 surface.

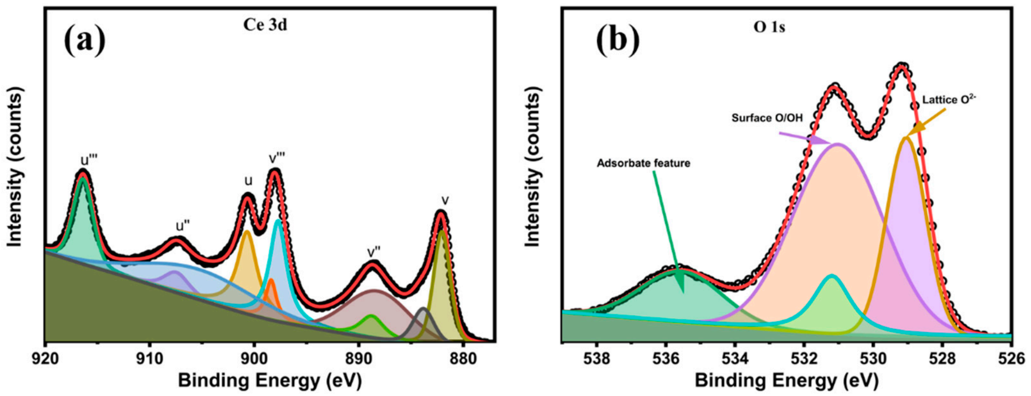

Figure S1. XPS analysis of CeO_2. (a) Core spectra of Ce 3d. (b) Core spectra of O 1s.

2. Kinetics of electrode material

Kinetic evaluation of the CV profiles using the relation $i(V) = k_1 v + k_2 v^{1/2}$ reveals that CeO_2, Y_2O_3, Sm_2O_3, and Pr_2O_3 exhibit strongly diffusion-dominated behavior at low scan rates, with diffusion contributions exceeding 93%. As the scan rate increases, the capacitive fraction rises because bulk ion diffusion becomes progressively limited. At 0.1 V s^{-1}, the surface-controlled contributions reach 31.88% for CeO_2, 33.28% for Y_2O_3, 40.56% for Sm_2O_3, and 37.39% for Pr_2O_3. In contrast, La_2O_3 displays markedly faster surface kinetics, with its capacitive share increasing from 39.99% at 0.001 V s^{-1} to 86.95% at 0.1 V s^{-1}. Overall, La_2O_3 shows the most pronounced surface-dominated charge-storage behavior, whereas the remaining oxides retain substantial diffusion-controlled contributions across the tested scan rates.

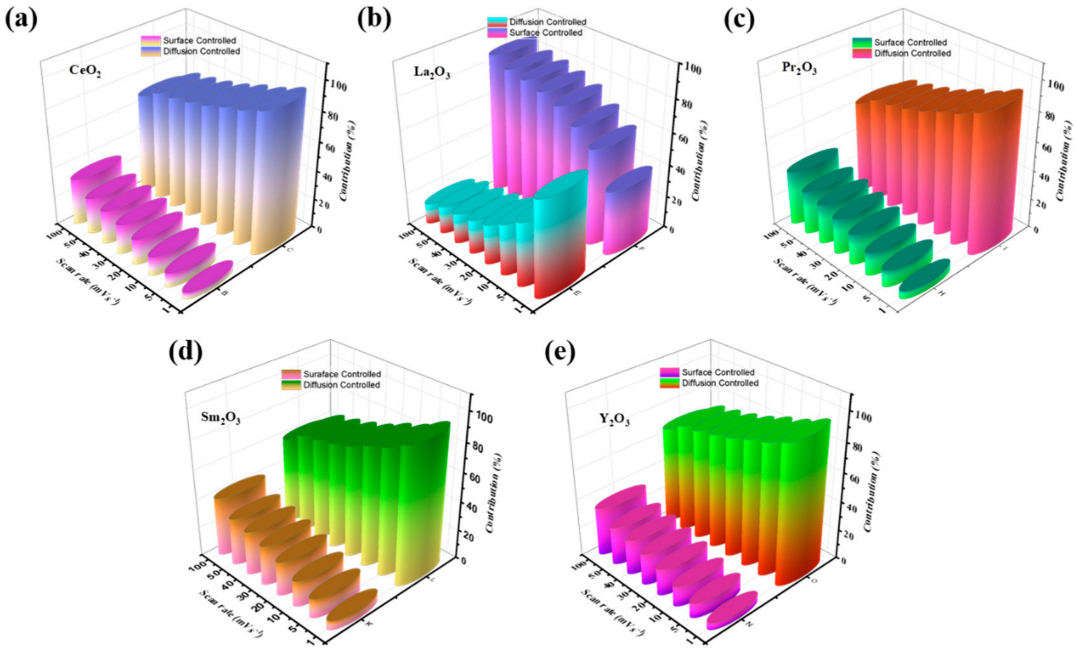

Figure S2. Relative contributions of different energy-storage mechanisms as a function of the scan rate.

Table S1. Comparative summary of the electrochemical performance of synthesized materials with the reported literature.

Material	Synthesis method	Electrolyte	Electrode performance	Device Performance	Ref.

CeO_2	Solvothermal method	3 M KOH	1801 Fg^{-1} at 1 Ag^{-1}	Energy density of 57.6 Wh kg^{-1} and a power density of 771 W kg^{-1}; 83% capacitance retention after 1000 cycles	[26]
La_2O_3	Chemical bath deposition	1 M KOH	147 Fg^{-1} at 5mVs^{-1}; 96% capacitance retention after 2000 cycles	NA	[27]
Y_2O_3-α-Fe_2O_3	Hydrothermal	3 M KOH	1216 Fg^{-1} at 1 Ag^{-1}	Energy density of 28.8 Wh kg^{-1} and a power density of 800 W kg^{-1}; 92.5% capacitance retention after 5000 cycles	[21]
CeO_2/MWCNTs	Chemical bath deposition	0.5 M NaOH	1215.7 Fg^{-1} at 2 mVs^{-1}; 92.3% capacitance retention after 10000 cycles	Energy density of 85.7 Wh kg^{-1} and a power density of 2.6 kW kg^{-1}; 92.1% capacitance retention after 10000 cycles	[36]
Bi_2Se_3-Sm_2O_3/PANI	Hydrothermal	1 M KOH	1399 Fg^{-1} at 5 mVs^{-1}; 91.75% capacitance retention after 5000 cycles	NA	[37]
CeO_2-Sm_2O_3	Hydrothermal	3 M KOH	397.5 Cg^{-1} at 10 mVs^{-1}	Energy density of 32.85 Wh kg^{-1} and a power density of 4223.91 W kg^{-1}; 86.92% capacitance retention after 3000 cycles.	[14]
Pr_2O_3/$C_{18}H_6Cu_3O_{12}$	Hydrothermal	1M KOH	2046 Cg^{-1} at 3 mVs^{-1}	Energy density of 67 Wh kg^{-1} and a power density of 6114 W kg^{-1}; 98 % capacitance retention after 3000 cycles.	[15]
CeO_2	Hydrothermal	3 KOH	684 Fg^{-1} at 1 Ag^{-1}	Energy density of 20 Wh kg^{-1} and a power density of 1000 W kg^{-1}; 100 % capacitance retention after 2000 cycles	Present study
La_2O_3			307 Fg^{-1} at 1 Ag^{-1}	NA	
Sm_2O_3			445 Fg^{-1} at 1 Ag^{-1}	NA	
Y_2O_3			288 Fg^{-1} at 1 Ag^{-1}	NA	
Pr_2O_3			501 Fg^{-1} at 1 Ag^{-1}	NA	
